# The Grip Concept of Incisional Hernia Repair—Dynamic Bench Test, CT Abdomen With Valsalva and 1-Year Clinical Results

**DOI:** 10.3389/fsurg.2021.602181

**Published:** 2021-04-14

**Authors:** Friedrich Kallinowski, Dominik Gutjahr, Felix Harder, Mohammad Sabagh, Yannique Ludwig, Vladimir J. Lozanovski, Thorsten Löffler, Johannes Rinn, Johannes Görich, Annette Grimm, Matthias Vollmer, Regine Nessel

**Affiliations:** ^1^General, Visceral and Transplantation Surgery, University Hospital Heidelberg, Heidelberg, Germany; ^2^General and Visceral Surgery, Gesundheitszentren Rhein-Neckar (GRN) Hospital Eberbach, Eberbach, Germany; ^3^General and Visceral Surgery, Kreiskrankenhaus Bergstrasse (KKB) Hospital Bergstrasse, Heppenheim, Germany; ^4^Radiological Center, Eberbach, Germany; ^5^Institute of Biomechanics, Hamburg University of Technology, Hamburg, Germany; ^6^General, Visceral and Pediatric Surgery, Klinikum Am Gesundbrunnen, Heilbronn, Germany

**Keywords:** bench test, computerized tomography, incisional hernia, GRIP, CRIP, hernia repair, hernia mesh, hernia mesh fixation

## Abstract

Incisional hernia is a frequent consequence of major surgery. Most repairs augment the abdominal wall with artificial meshes fixed to the tissues with sutures, tacks, or glue. Pain and recurrences plague at least 10–20% of the patients after repair of the abdominal defect. How should a repair of incisional hernias be constructed to achieve durability? Incisional hernia repair can be regarded as a compound technique. The biomechanical properties of a compound made of tissue, textile, and linking materials vary to a large extent. Tissues differ in age, exercise levels, and comorbidities. Textiles are currently optimized for tensile strength, but frequently fail to provide tackiness, dynamic stiction, and strain resistance to pulse impacts. Linking strength with and without fixation devices depends on the retention forces between surfaces to sustain stiction under dynamic load. Impacts such a coughing or sharp bending can easily overburden clinically applied composite structures and can lead to a breakdown of incisional hernia repair. Our group developed a bench test with tissues, fixation, and textiles using dynamic intermittent strain (DIS), which resembles coughing. Tissue elasticity, the size of the hernia under pressure, and the area of instability of the abdominal wall of the individual patient was assessed with low-dose computed tomography of the abdomen preoperatively. A surgical concept was developed based on biomechanical considerations. Observations in a clinical registry based on consecutive patients from four hospitals demonstrate low failure rates and low pain levels after 1 year. Here, results from the bench test, the application of CT abdomen with Valsalva's maneuver, considerations of the surgical concept, and the clinical application of our approach are outlined.

## Introduction

The occurrence of an incisional hernia indicates the development of a weakness of the sutured abdominal wall caused by mechanical overload, defective wound healing, and/or inadequate scar formation. Patients with an incisional hernia often complain of pain and a gradually increasing hernia size indicating an overstretching of the tissues. The rates of recurrences are still unacceptably high. Some of the main reasons why incisional hernia recur in such high incidence is the inappropriate selection of the adequate mesh, its size and its fixation according to the size of the hernia. Also, abdominal wall elasticity plays an important role, which is rarely examined preoperatively. How can a durable, long-lasting repair of incisional hernias be designed? We wanted to gain insight into the potential of cyclic loading and shakedown analysis for incisional hernia repair.

**Figure 1:**
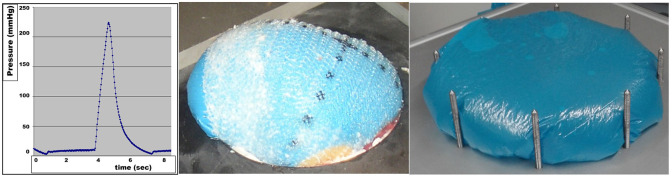
DIS impact without a plateau phase **(left)** registered every 30 ms, the balloon displacing an hernia mesh **(middle)** and the mushroom cap-like protrusion of a DIS test with a plateau phase **(right)**.

**Figure 2:**
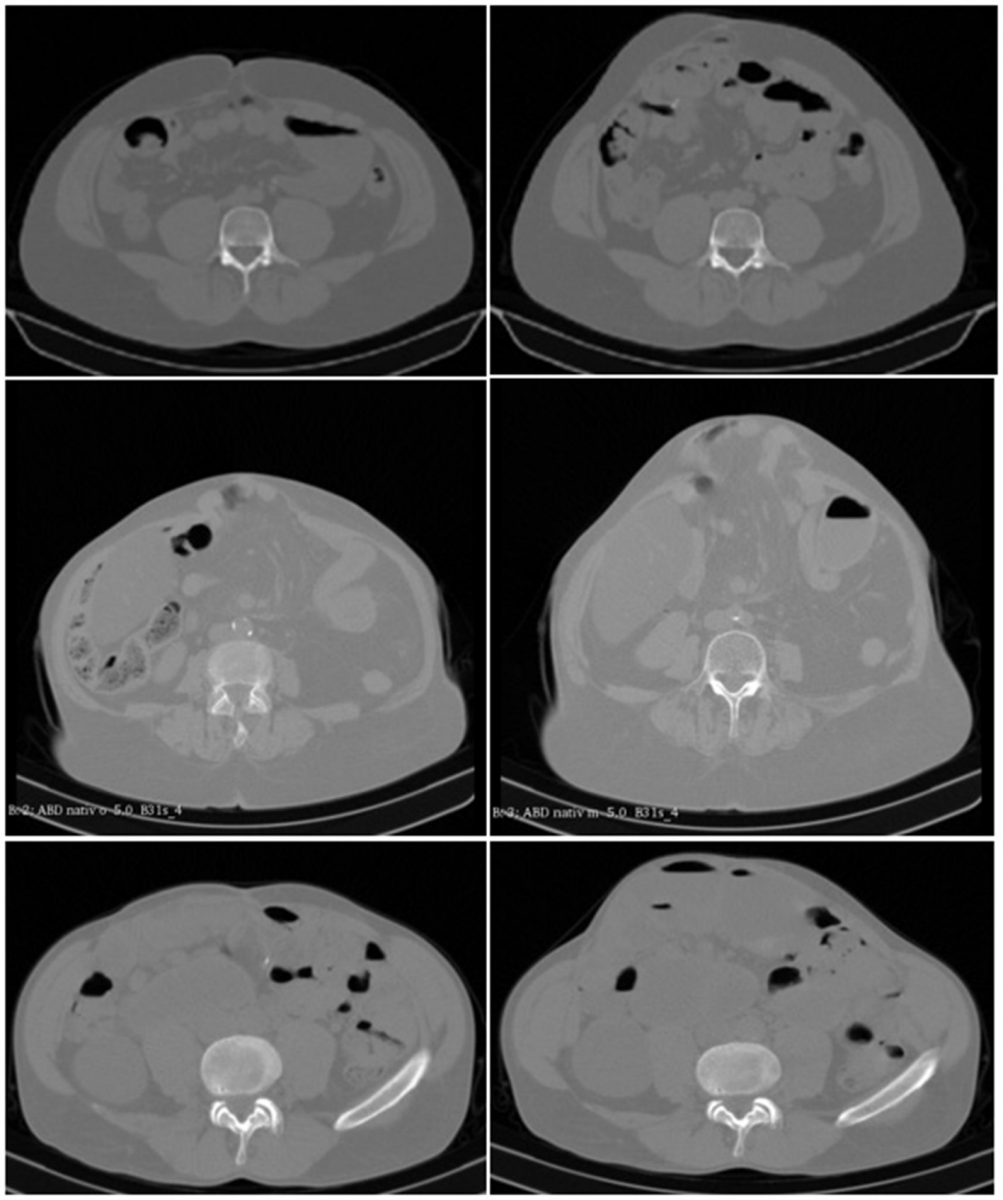
**(Top)** CT scans of the abdomen without contrast medium of a 35-year-old male with an incisional hernia after liver transplantation at rest **(Left)** and during Valsalva's maneuver **(Right)**. It can be noted that the lateral musculature contracts, and the abdominal contents bulge forward with a distension of the hernia opening by about 30%. **(Middle)** CT scans of the abdomen without a contrast agent of a 73-year-old woman with an incisional hernia after pancreatic resection at rest **(Left)** and during Valsalva's maneuver **(Right)**. It can be seen that the anterior abdominal wall bulges forward, and the hernia sack increases in size without enlargement of the hernia opening. **(Bottom)** CT scans of the abdomen without added contrast of a 62-year-old man with an incisional hernia after laparostoma and short bowel syndrome resulting from multiple intestinal fistulae after an ileus. The functional state at rest **(Left)** and during Valsalva's maneuver **(Right)** corresponds to the two upper rows. It can be recognized that the left lateral musculature is displaced by the abdomen bulging forward opening the hernia base by about 50%. (we acknowledge the help of Samuel Voss in the selection of scans in the upper and lower row).

We conducted a feasibility study to apply continuum biomechanics to incisional hernia repair. A bench test for cyclic loading was developed to characterize the impact-related biomechanics of materials used for repair. Dynamic intermittent strain (DIS) resembling coughs can characterize any influence numerically with relative figures (1, 2). The results can be summed up to indicate the critically needed (CRIP) and the gained resistances to impacts delivered by pressure (GRIP) before and during surgery (3). The CRIP gives a threshold for the reconstruction to survive 425 repeated impacts within a period of 4 h on the bench test. The GRIP includes the calculation of a relative value characterizing the retention force of a mesh with its fixation elements at the mesh–tissue interface. We believe that a durable reconstruction requires the GRIP to be higher than the CRIP value.

Computerized tomography of the abdomen at rest and during Valsalva's maneuver gives insight into the overall shift of organs and tissues upon strain and permits the analysis of tissue elasticity preoperatively (4, 5).

We applied the concept to consecutively treated patients with incisional hernia as a prospective observational registry study. As the main outcome parameter, recurrences were reported after 1 year. As secondary endpoint, pain levels were observed for 1 year.

## Materials and Methods

We conducted a feasibility study for a novel biomechanical approach involving a self-built bench test, a CT evaluation developed by our group, and a clinical application based on the worldwide largest hernia registry Herniamed®. We wanted to gain insight into the potential of cyclic loading and shakedown analysis for incisional hernia repair.

The study consists of three steps with each one being a prerequisite of the next step. Without a bench test, enabling cyclic loading shakedown cannot be tested for. Without coefficients derived from the bench test characterizing each influence, individualization is neither possible nor necessary on a scientific basis. Without criteria for individualization, meshes and surgical techniques cannot be tailored. CT scans at rest and with strain demonstrate large interindividual differences in elasticity. Tissue elasticity is the major influence on the bench test. Therefore, individualization is necessary. The prospective observational registry study shows the potential of the approach with each element influencing the design and contributing to the surgical execution of incisional hernia repair.

### Description of the Bench Test

The bench test uses hydraulically driven repeated sharp impacts resembling coughing actions (Figure 1). Since the intermittent strain varies dynamically every 4–6 s, the term dynamic intermittent strain (DIS) test was used for the application (1, 6). This self-built DIS test was designed in two versions. In the first version, a protruding balloon bulged as a ball. Upon the impacts, a creeping motion of an hernia mesh was observed leading to dislocation of the mesh in about 85% of the reconstructions investigated. In the second version, a polyethylene foil was used as a stamp with a wider contact surface similar to a mushroom cap for the dynamic deliverance of energy to the tissues resulting in comparable effects. Both designs were computer controlled to simulate a coughing or a sharp straining action with <1 s to reach the peak and a relaxation time up to 3 s followed by a resting time of 2 s.

Commercially available hernia meshes and fixation systems such as sutures, tacks, and glue were tested on a self-built bench test (3). Two different tissues were investigated with differences in their elasticity: beef flank was found to be more distensible and to bear more load compared with pig belly (5, 7). Hernia sizes from 5 to 12.5 cm in diameter were investigated in detail with two different mesh positions: sublay/retromuscular and underlay/IPOM. In order to investigate the different mesh positions, meshes were placed between the respective tissue layers. From the results, meshes were classified according to the need for fixation (DIS classes A–C) (8). Fixation devices were graded according to the retention force per single element or, in case of glue, per square centimeter.

At rest, the tackiness of the mesh sticks the textile to the tissue surface. Meshes with high tackiness are less likely to need fixation. The basal pressure of the DIS machine increases the stiction between tissue and textile. Fixation is added as spot or area gluing, as tacks of various kinds or as running or single-knot sutures. Fixation increases the retention force. The pulse transmits energy to the reconstruction, elongates tissue and textile to a different extent, and causes in this way a sliding or creeping force counteracting the retention force. If the reconstruction fails in one spot, failure is likely to occur on the bench test. The results obtained should be reproducible even under unstable conditions.

### Consideration for the Clinical Application of the GRIP Concept

In order to assess this balance of power and to address the various influences, a numerical value was derived. This value describes the retention force of the whole reconstruction. The surgeon can use the value to tailor the procedure to the needs of the individual patient. Starting from the mesh-defect-area-ratio (MDAR), such a numerical value was developed (9, 10). We choose to multiply the MDAR with coefficients. According to a general description, a coefficient is a multiplicative factor in some term of a polynomial, a series, or any expression; it is usually a number, but may be any expression. In the latter case, the variables appearing in the coefficients are often called parameters and must be clearly distinguished from the other variables (Coefficient—Wikipedia). We choose parameters that characterize the tackiness of the mesh, the strength of the fixation, the position of the mesh, the elasticity of the tissues, and other influences. The coefficients available so far are given in Table 1.

**Table 1:** Influences to be considered as coefficients during the planning of the reconstruction.

Tissue distension	1–18
Meshes	0.1–1.44
Sutures, Securestrap^®^	0.4–0.6
Absorbatack^®^, Glubran^®^, Tisseel^®^	0.15–0.3
Position in the abdominal wall	0.9–1
Peritoneum closure factor	4

*Please note that the values are derived with the bench test described above from about 200 different reconstructions, which were selected from about 4,500 possibilities. The mesh position was investigated with the same meshes and gave slightly different gripping coefficients so far. The fixation coefficients were calculated per suture knot loop, per tack, per fixation point or per gluing area of half a square centimeter. For the individual surgical unit, the material preferentially used should be tested before coefficients are applied (3)*.

The calculations with the coefficients are given in formulas (1) and (1a) and return the gained resistance to impacts related to pressure (GRIP) in relative numbers (3). The **g**ained **r**esistance of a reconstruction toward **i**mpacts delivered by **p**ressure was shortened to the acronym GRIP as detailed previously (8).

(1) $$\begin{array}{l} \text{Grip}=\text{MDAR}*\text{bonding factor}\\ \text{ + peritoneum closing factor} \end{array}$$

This value was found useful to assess several 100 different reconstructions so far. The formula has been expanded to include other influences as coefficients as well.

(1a) $$\begin{array}{l} \text{Grip}=\text{MDAR}*\text{meshbonding factor}*\text{fixationbondingfactor}\\ \;*\text{mesh position factor + peritoneum closing factor } \end{array}$$

Due to ongoing research efforts, it is likely that more coefficients will be included in the future. Missing is a shape factor to better describe the geometry of the defect or the shape of the mesh. The mesh overlap may be detailed with an overlap factor characterizing a retention force needed on the edge. Please note that the coefficients for the various factors have to be determined for each condition with a bench test several times before the factors can be used to plan a reconstruction.

For a given mesh, it is possible to describe conditions of 100% stability in a DIS test as a **c**ritical **r**esistance of **i**mpacts delivered by **p**ressure shortened to the acronym CRIP (3).

The CRIP value can be calculated before the surgical procedure as:

(2) $$\begin{array}{r} \text{CRIP }=\;0.5\text{∗hernia size}+15 \end{array}$$

according to (3).

Data on the bench test indicate that the tissue elasticity is an important modulator of the CRIP value. With very lax tissue, higher CRIP values are required for stability.

For the clinical application of the GRIP concept, it is not sufficient to transfer the bench test data to the surgical work. The defect area and the tissue elasticity have to be determined in the individual patient. Since the defect area can vary with pressure and muscular contraction, it has to be determined in at least two states—at rest and under load. The same holds true for the tissue elasticity. From the clinical options available, we choose to perform a CT scan of the abdomen at rest and during Valsalva's maneuver.

### CT Abdomen at Rest and During Valsalva's Maneuver

The protocol for CT scanning was adapted from a low-dose, no-contrast protocol implemented for symptomatic kidney stones. All CT imaging data were collected twice without contrast medium during deep inspiration. Following the scout for planning, the CT acquired data sequentially (slice thickness 0.6 mm, 110–130 kV). The whole abdomen from the diaphragm to the symphysis was scanned: first in relaxation with the abdomen at rest. As a second step, the patient strained oneself with Valsalva's maneuver. Examination time was <30 s using a Somatom® 16 Scanner (Siemens, Erlangen, Germany).

Examples are given in Figure 2. The data were evaluated as described previously (4, 5, 11).

### Applying the GRIP Concept to the Individual Patient

**Figure 3:**
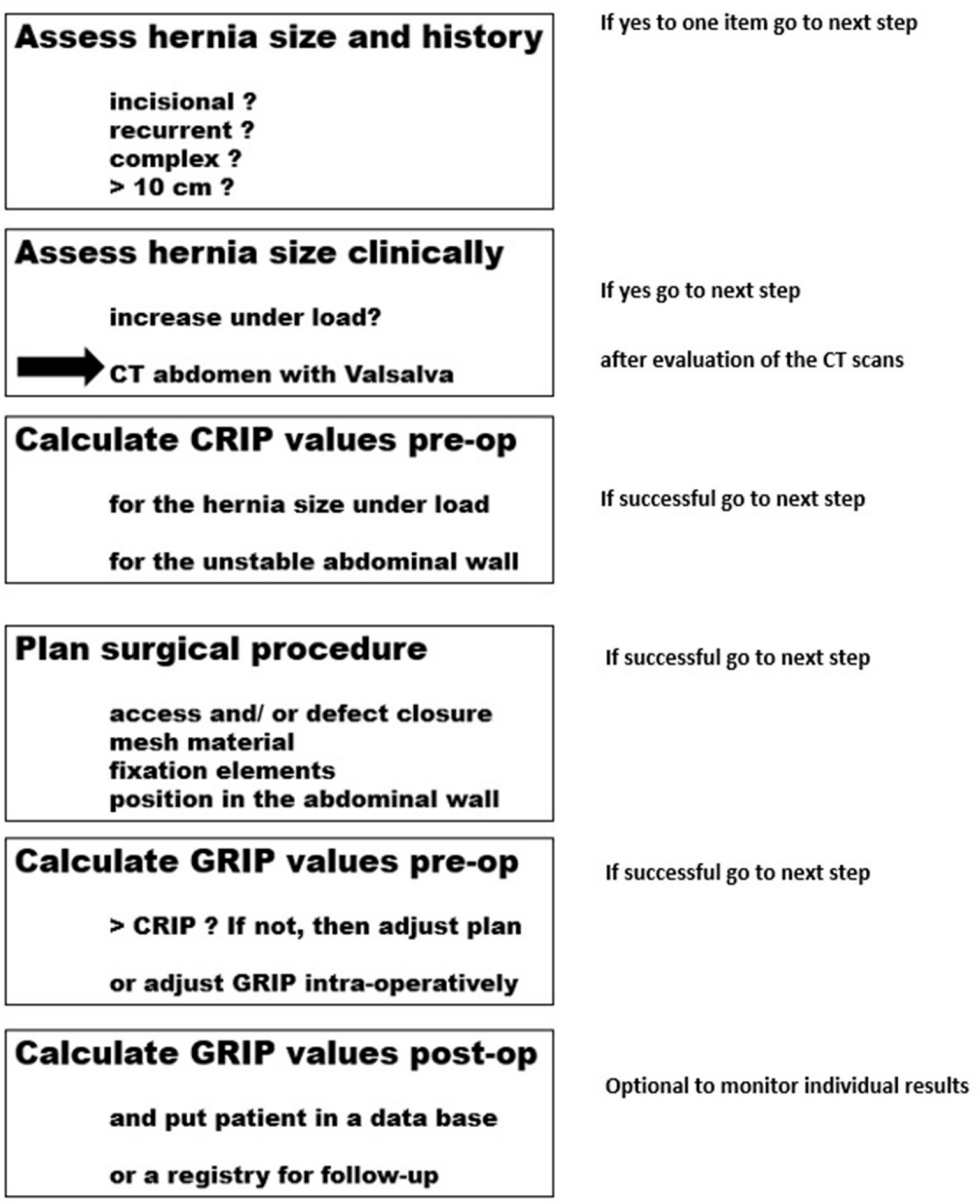
Considerations for the surgical implementation of the GRIP concept.

The GRIP concept was applied to patient care to reduce complication and recurrence rates after incisional hernia repair. The individual patient was evaluated according to the flow chart depicted in Figure 3. More complex hernia cases and/or patient with many comorbidities are primarily eligible for evaluation. Surgeons in the STRONGHOLD group decided to apply the GRIP concept to smaller hernia sizes for training purposes.

If the hernia size is found to distend markedly on clinical observation, or if more than one incision was present on the abdominal wall indicating a battlefield abdomen, a computed tomography of the abdomen at rest and during Valsalva's maneuver was performed, and the tissue elasticity was evaluated as described above. The tissue elasticity was multiplied with the CRIP value calculated.

The reconstruction was planned to surpass the elasticity-adjusted CRIP value. The planning of the reconstruction started with an estimate on the desired MDAR. According to Kallinowski et al. (8), a DIS class A mesh gives the best retention force due to a high mesh bonding factor as detailed in formula (1a) above. Both DIS class A meshes used here have high retention forces with Progrip® exhibiting a coefficient of 1.44, Dynamesh® Cicat one being 1.0 (10). The bench test is used to continuously test new materials and to derive the respective coefficients. New results are presented in a monthly weblog, www.hernia-today.com [Influences on the GRIP calculation—Hernie heute (hernie-heute.com)]. The variation of the coefficients available so far is summarized in Table 1.

During the planning of the surgical procedure, the GRIP values are adjusted and recalculated selecting other meshes, alternate sizes, different fixation schemes, and so forth until the planned GRIP value is larger than the required CRIP with the desired safety margin. This planning procedure profoundly influences the surgical procedure to be performed. Since the retromuscular position gives the better retention force compared with an IPOM procedure, all repairs intended to place the mesh before the peritoneum behind the musculature. A total of 72 sublay positions with six MILOS approaches and four eMILOS approaches were attempted. A transversus abdominis release was planned in 19 cases. In these cases, peritoneal flap techniques were generally included in the plan to provide large areas for tension-free repair, embedding the required mesh sizes and preventing increased intra-abdominal pressures at the same time. Preperitoneal underlay mesh placements (PUMP) using flipped Progrip® meshes with the lactic acid grips oriented toward the musculature were included in the planning in four patients.

After surgery, the particulars of hernia and mesh sizes and shapes, the kind and number of fixation elements, the position of the mesh within the abdomen, and the closure of the peritoneum are noted and entered into the STRONGHOLD/Herniamed® registry. All patients received a telephone interview after 1, 6, and 12 months. If the patients complained of pain or a protrusion, a clinical investigation was followed by an ultrasound, magnetic resonance imaging, or a CT scan as needed.

### The Stronghold/Herniamed® Registry

Within the Heidelberg surgical community, 96 patients were consecutively treated by 10 different surgeons in four hospitals. The data were included in the Herniamed® registry, which was expanded with a data sheet called STRONGHOLD. Within STRONGHOLD, seven additional items have to be reported taking about 1 min with an algorithm calculating MDAR and GRIP thereafter. We report results after 1 year for the first 96 consecutive patients. The patients were followed by telephone. If pain or bulging was reported, ultrasound and/or CT scans of the abdomen at rest and during Valsalva's maneuver were performed. Pain was rated from zero to 10 according to the commonly used numerical or visual analog pain scales.

The approach reported here is limited by the definition of the load case, the missing load-limit curve, and the lack of a control group. A better definition of the load case requires the analysis of critical confounders in a large cohort, e.g., by propensity score matching. The load-limit curve is evaluated for elastic tissue assessing the peak pressure and the length of the plateau phase. Preliminary results show the length of the plateau phase to be more influential. A control group requires the expansion of the data base, e.g., by randomization or—more cost efficient—by propensity-score matching again.

### Statistical Analysis

Data were collected in Excel spreadsheets. The results were depicted with box-and-whisker-plots and as time lines. Descriptive statistics were calculated as needed. The Kruskal–Wallis test was used for the assessment of group differences. Differences were considered significant after a Bonferroni correction on an error probability of 1%. In case of significant group differences, u-tests for non-paired observations were applied to find differences pairwise.

## Results

### Influence of the Observer on the Results of the Bench Test

**Figure 4:**
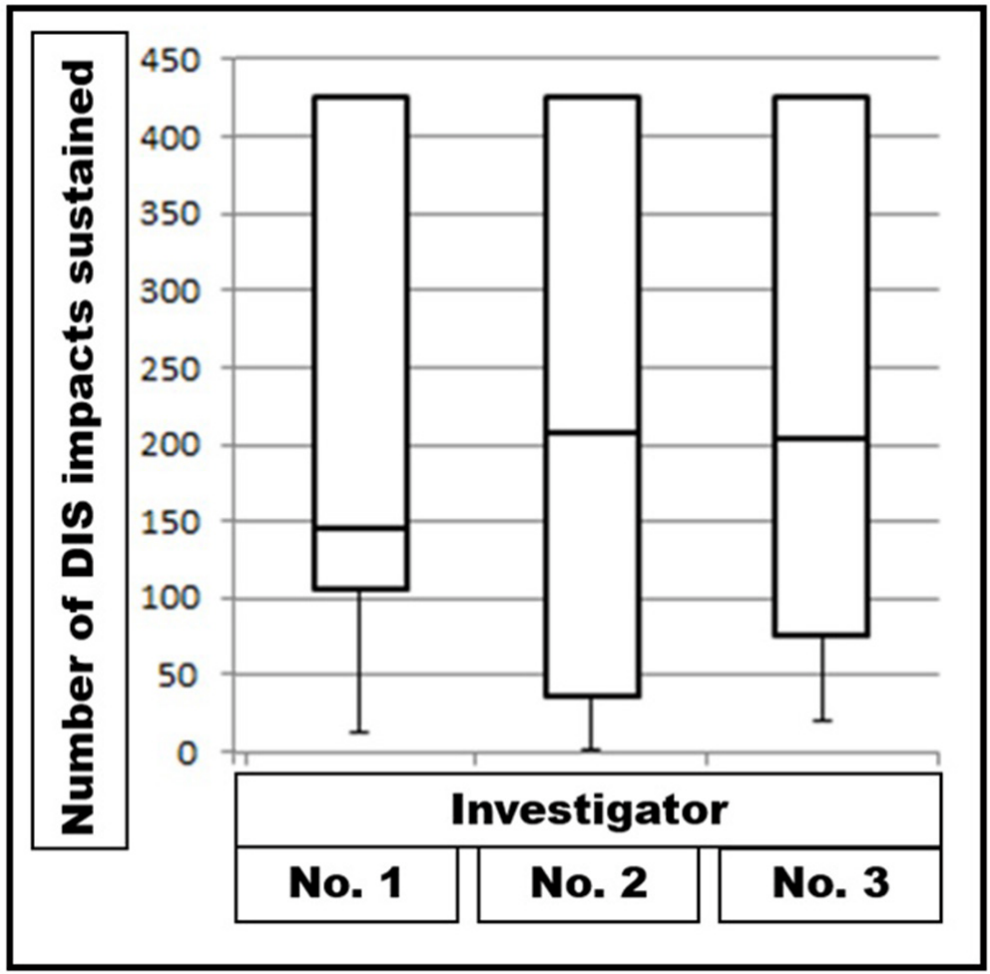
Box-and-whisker plots of DIS impacts sustained by Dynamesh® Cicat hernia mesh implanted under unstable conditions by three different investigators performing series with 10 different experiments each under the same conditions. The differences are not significant (*p* = 0.96079).

No influence was found in repeated experiments as long as the baseline pressure was kept above 4 and below 10 mmHg (Figure 4). For these experiments, a setting with 50% stability for 450 DIS impacts was chosen. To reach this aim, a 12.5-cm round Dynamesh® Cicat bridged a round defect with a diameter of 5 cm without fixation. A variation of about 10% of the median values was noted during the repeated series with 10 experiments each.

### Preoperative Assessment of the Tissue Elasticity With CT Abdomen at Rest and During Valsalva's Maneuver in Individual Patients

**Figure 5:**
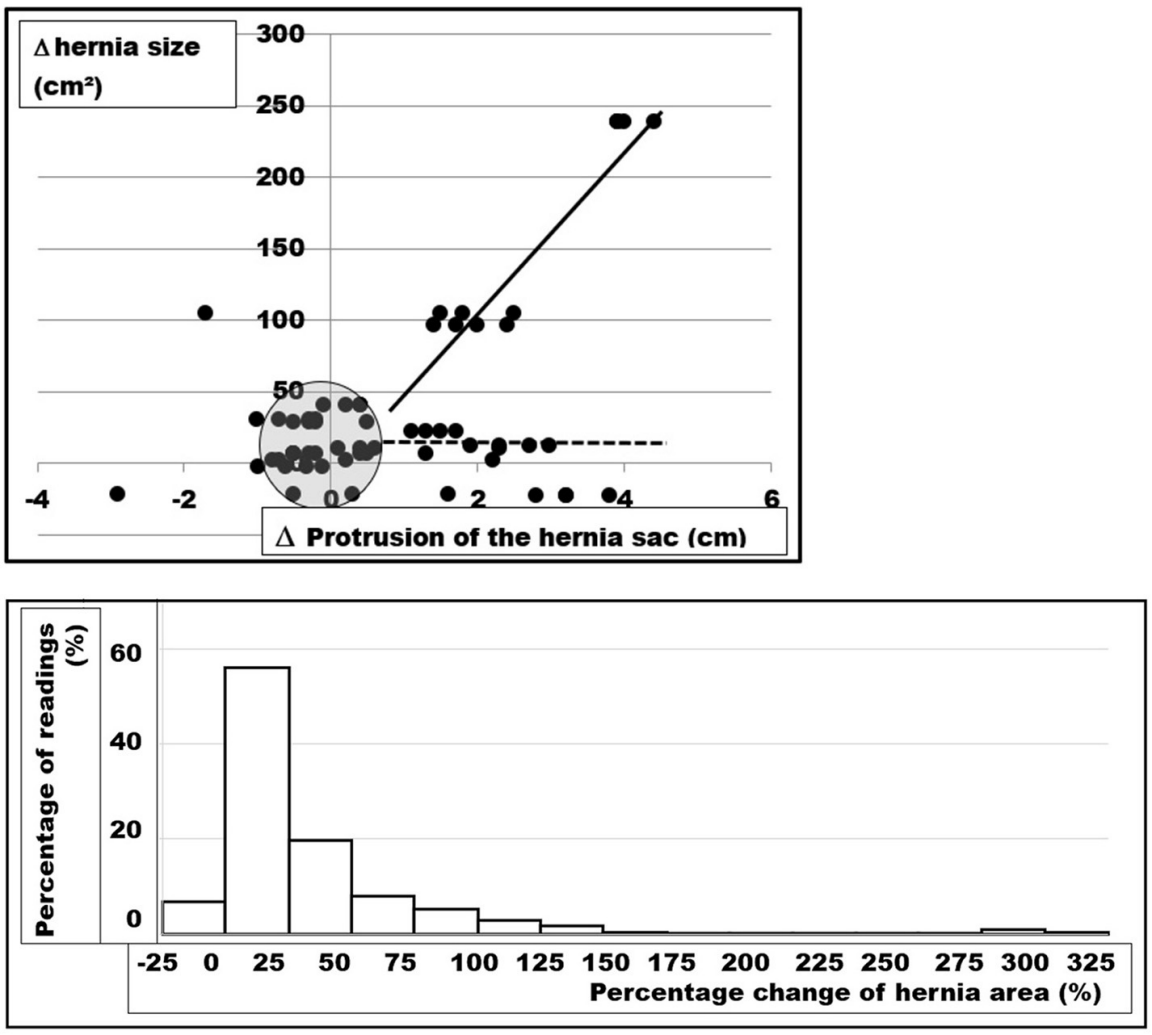
**(Top)** Changes in the hernia size as a function of the distension of the hernia sac upon Valsalva's maneuver of five patients. The CT scans of five patients were analyzed four times by three different investigators giving a total of 12 readings from each CT abdomen. The evaluation procedure and the interobserver variation has been described previously (4). About half of both parameters change <10% (dots in the shaded circle). In about one quarter, the hernia sac expands with the hernia opening staying almost constant (dotted line). In about one fifth, both the hernia defect and the hernia sac dilate (solid line). In a few readings, the musculature contracts the hernia sac with unpredictable behavior of the hernia size (left side of the illustration). **(Bottom)** Frequency distribution of measured changes of the hernia area from CT scan of the abdomen at rest and during Valsalval's maneuver of 67 patients analyzed in this manuscript. Each patient was analyzed one to four times by three to six observers giving a total of 253 readings. Marked variation is obvious with most values ranging between no dilatation and 150% enlargement. About half of the hernia areas change <25% in size upon Valsalva's maneuver.

Preoperatively, the elasticity of the abdominal wall of the individual patient was assessed by three to five investigators at least three times as previously described (3, 4). The results are depicted in Figure 5 for illustration.

### Applying the GRIP Concept to the Individual Patient

Preoperatively, the type of mesh and its size were chosen to reach the desired MDAR. The surgical strategy was planned, and several alternatives to reach GRIP > CRIP were calculated. Intraoperatively, the size of the mesh was adopted to the anatomical findings with an elliptic, round, or square shape as desired. Changes in the hernia and/or the mesh sizes were followed by a recalculation of the MDAR and the number of the fixation elements. The retromuscular space was limited in the sublay area and required a posterior component separation for larger hernia areas in an additional 10 cases to the 19 patients already planned in this way.

During the surgical performance, anatomical variations or technical aspects necessitated modifications of the planned procedures in 12 cases. In addition, the fixation factor was varied in the majority of cases after intraoperative assessment of the regional instability of the abdominal wall. Sutures were augmented with tacks in 39 cases to safe operation time.

### Clinical Application of the Biomechanical Concept

A total of 96 patients were consecutively operated on between July 1, 2017 and July 31, 2019 in four different hospitals. Demographic and comorbidity data are given in Table 2. Half of the patients were still professionally active (mean age ± SD: 62 ± 13; range: 27–92).

**Table 2:** Demographic data and comorbidities of the 96 patients reported here.

	***N***	**%**
Female	51	53
Male	45	47
ASA 1	8	8
ASA 2	42	44
ASA 3	45	47
ASA 4	1	1
Average body height (m)	1.71	
Average body weight (kg)	85	
Average BMI (kg/m^2^)	29	
Bleeding disorder/ anticoagulation drugs	37	39
COPD, active smoker	31	32
Diabetes mellitus, not or badly controlled	16	17
concurrent chemotherapy for carcinoma or steroid therapy	12	13
Immunosuppression, liver and/ or kidney transplant recipient	11	11
Renal insufficiency or dialysis	5	5
Aortic aneurysm >4 cm in largest diameter	4	4
Morbid obesity	3	3
Badly controlled hypertension	2	2
Pulmonary embolism	2	2
Immobilizing spine fracture	1	1
portal vein thrombosis	1	1
Bone tuberculosis	1	1
Immunoglobuline A deficiency	1	1
Antibiotic therapy for urinary infection	1	1

*ASA indicates the risk classification of the American Society of Anesthesiologists. N means the number of patients*.

The indication for surgery was enlargement of the hernia during time in all cases and disabling pain in 39 patients. Only one emergency case was noted. Primary hernia was prevalent in 78% with 21 recurrent cases (17 first, one second, three fourth recurrence). The width of the hernia was below 5 cm in 18 cases, between 5 and 10 cm in 49 patients, and above 10 cm in 29 orifices. Median hernia openings were noted in 64, purely lateral orifices in 14 cases. Combined median and lateral hernias occurred in 18 cases.

Median hernia size was 39 cm^2^ (mean: 82 ± 94; range: 2–491). In almost all cases, DIS class A meshes were implanted (43 Dynamesh® Cicat, 41 Progrip®, one Ultrapro®, and one Proceed®). Larger hernia sizes were cared for with Dynamesh® Cicat (median hernia size: 113 cm^2^; mean: 132 ± 108 cm^2^; range: 12–491 cm^2^). Median mesh overlap was 4.5 cm (mean: 4.7 ± 1.7; range: 1.5–10) before closure of the defect, which was attempted at least of the anterior wall in all cases using tissue flaps as needed. This was mainly due to Progrip® needing smaller overlap on bench testing for durable repairs due to its 44% higher gripping power (median Progrip® overlap: 4.5 cm; median Dynamesh® Cicat overlap: 5 cm). Meshes were cut to an elliptical shape in 53 cases, being left square in 41 patients. Two hernia defects were covered with round hernia meshes. In general, the posterior wall was closed (89 cases) with 86 absorbable and two permanent suture materials. In seven cases, the hernia sac was redressed, and the defect was bridged. In one case, a sandwich was constructed with Phasix®ST augmentation. Median CRIP was calculated as 37 (mean: 57 ± 48; range: 16–260). The median MDAR was 9 (mean: 14 ± 20; range: 2–143). The reconstructions were augmented with a median of 20 fixation points (mean: 38 ± 41; range: 0–257). Fixation was mostly done with non-absorbable sutures (Prolene® 2-0 in 79 cases). For additional support, tacks were used in 44, pull-out sutures in five, and fibrin glue in one case. Median GRIP was 108 (mean: 161 ± 156; range: 14–928). On the average, GRIP was three times the required CRIP value. Details are given in Table 3. Median theater time was 127 min (mean: 143 ± 68; range: 47–360 min).

**Table 3:** Intra- and postoperative details on the mesh position, the surgical procedure, and intra- und postoperative complications.

**Mesh position/surgical procedure**	**Cases**		**Complication**					
	**Number**	**%**	**Intraoperative**			**Postoperative**		
			**Bowel**	**Bladder**	**Bleeding**	**Wound**	**Seroma**	**Pneumonia**
Retromuscular	66	69	1	1	1	1	2	0
Posterior release	29	30	2	0	0	2	2	1
IPOM	1	1	0	0	0	0	0	0
MILOS open	6	6	0	0	0	0	0	0
MILOS endoscopic	4	4	0	0	0	0	0	0

The postoperative complications held the patients for 7, 8, 9, 18, 19, and 113 days in the hospital with two patients suffering from two complications. No mortality was observed during the hospital stay or within 30 days thereafter.

**Figure 6:**
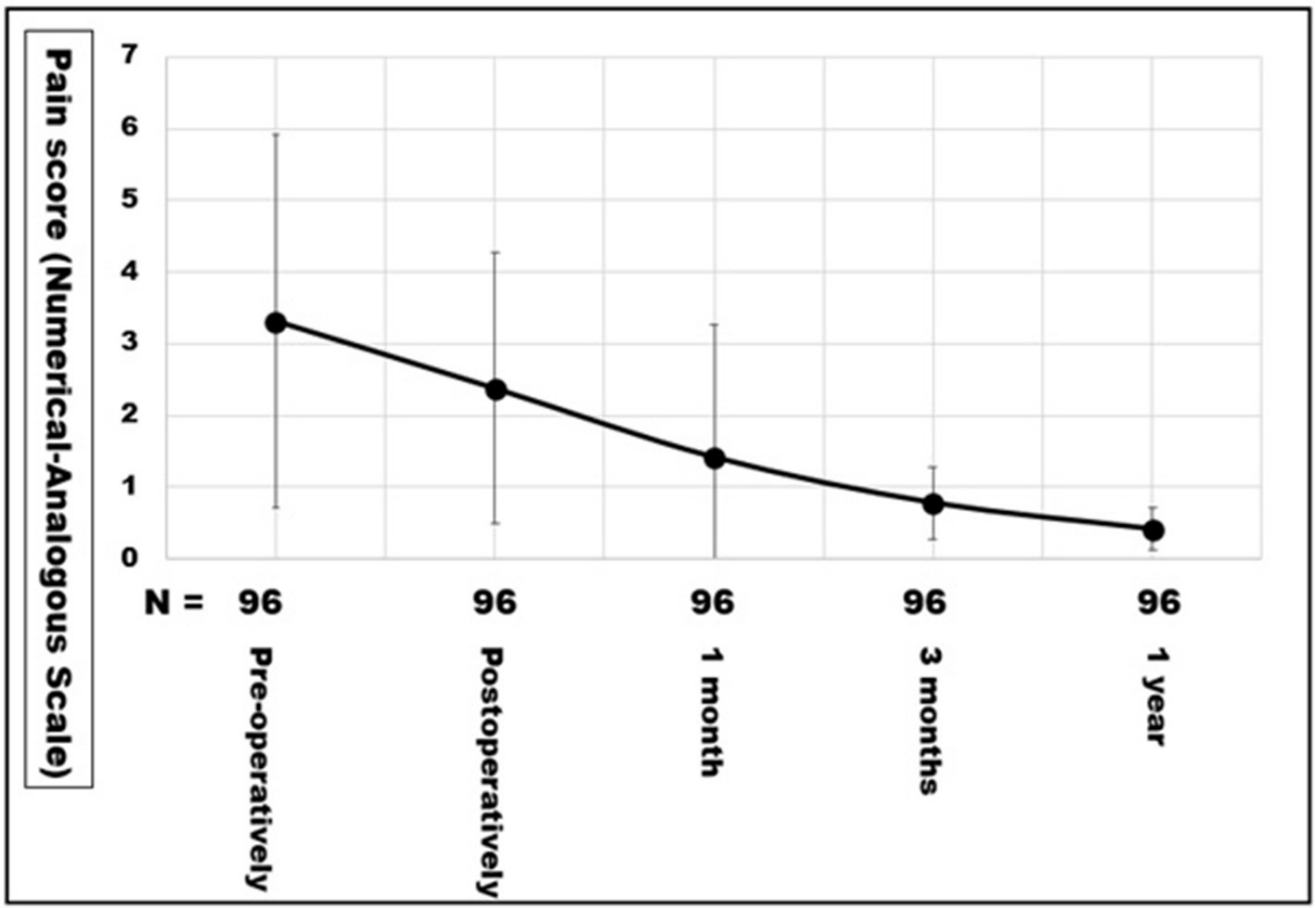
Pain levels as numerical analogous scale of the first 96 patients operated on between June 2017 and August 2019. The points indicate the means, the error bars the standard deviation.

In patients who reported a protrusion on follow-up, an ultrasound examination (*N* = 40) and/or a CT scan of the abdomen at rest and with Valsalva's maneuver were performed (*N* = 18). No recurrence of the repaired incisional hernia was detected so far with a CT scan of the abdomen including Valsalva's maneuver. One distant abdominal wall hernia after metachronous open cholecystectomy in another hospital, one pronounced rectal diastasis after a weight gain of 25 kg, and two inguinal hernias were diagnosed. Pain levels between NAS levels 1–5 were reported after 1 year by four patients after load bearing in excess of 2 h, none of which required a prescription for pain relief (Figure 6).

## Discussion

First, we show that bench tests for cyclic loading can be built (Figure 1). Over time, material coefficients can be accumulated (Table 1). The data facilitate a biomechanical approach to incisional hernia repair (Figure 3).

### Contribution of the Dynamic Bench Test to Durable Incisional Hernia Repair

Because the biomechanical behavior of the abdominal wall is complex, there is a need for an experimental approach to advance surgical science (12, 13). A self-built bench test permits the analysis of tissues, hernia meshes, fixation devices, and surgical techniques (3). During the bench test, a model hernia repair is subjected to repeated submaximal dynamic impacts simulating coughs. Plastic deformation of a newly formed compound can take place under these conditions. The deformation results in a shakedown of the structures to bear load or in a breakdown once the load limit is exceeded (14). The data from the bench test are the first application of the concept to incisional hernia repair. Using the data, a measure for dynamic friction called grip can be derived (8).

DIS loading in our bench test puts energy into the incisional hernia repair tested (Figure 1). The retention force of the mesh–tissue interface can be assessed in relative terms. So far, data on Progrip®, Dynamesh® Cicat and IPOM, Ultrapro®, TiMesh® light, PhysioMesh®, and Permacol® have been published with Progrip® and Dynamesh® Cicat classified as DIS class A meshes (Table 1) (8). The gripping factor of Progrip® was assessed as 1.44 that of Dynamesh® Cicat being 1.0.

Meshes and other hernia repair material are brought to the market after proof of the mechanical stability, harmlessness to living tissues, and longevity under physiological conditions (15, 16). Stress tests of the compounds made with various tissues, meshes, and fixation material have rarely been performed. It has been demonstrated that the wet compound requires a healing period to gain stable conditions (17). Networks from collagen fibers strengthen following cyclic loading (18). Phantom studies depend on the stretch ratio, the stress levels, and other influences (19). Freshly formed collagen fibers need crosslinking for shakedown (20). Since bending can already displace a mesh by several centimeters, a critical load limit has to be surpassed for stability (21). We defined this limit as coughing 425 times with intra-abdominal pressures above 150 mmHg. From published results and our own observations, about one third of our patients pass this threshold in the first 24 h after surgery (1, 22, 23). The GRIP and CRIP concepts can factor in all aspects mentioned above (8). We conclude that the bench test permits the assessment of the biomechanical properties and possible interactions of tissues and repair materials under pulse load.

### Influence of the Observer on the Results of the Bench Test

With the DIS test, the biomechanical properties of each part of an incisional hernia repair can be analyzed independently (Table 1). Critical parts of the compound can be identified. The results of the DIS test are reproducible (Figure 4). The interobserver variation can be estimated as 10–12%. For the clinical application, the variation of 10% should be built into the safety margin. During the planning procedure, the intended GRIP value should be at least 10% higher than the estimated CRIP (Figures 2, 3).

### Preoperative Assessment of the Tissue Elasticity With CT Abdomen at Rest and During Valsalva's Maneuver in Individual Patients

Tissues differ due to age, obesity, comorbidities, collagen composition, and other influences. The hernia orifice varies its position and its width within the abdominal wall. The muscular strength as a stabilizing factor and the unstable , debris-like zone surrounding the hernia orifice differ from patient to patient. Although tissue quality influences the biomechanics to a large extent, a paucity of data is available with no randomized trial reporting this item (24). Our attempt with Valsalva's maneuver during a CT scan of the abdomen gives a clue to the tissue elasticity of the individual patient. In about half the patients, the elasticity of the hernia sac and of the tissue surrounding the hernia orifice differs. The individual tissue elasticity can be considered and accounted for with the GRIP concept (3).

The conventional evaluation by man-based segmentation demonstrates high inter- and intraobserver variation (Figures 2, 5). This is mainly due to the subjective differentiation between the hernia area and the debris zone surrounding it (4, 5). The debris zone may involve scar formation, neuronal deprivation, or muscular wasting. The consequence is an anisotropic load distribution with potential for a regional instability leading to a recurrence. It is a commonly held belief that the size of an incisional hernia can be reliably given after a single assessment. Our work demonstrates that about 12 readings are necessary to bring the variation below 5% in cases of pronounced instability. Our practical solution involves multiple readings by different observers. New approaches to reach the area to be repaired more precisely and less time consuming involve artificial intelligence and non-rigid b-spline registration from CT scans done on the individual patient preoperatively (11).

Thirty-one randomized trials were analyzed, and only 14 reported the average hernia defect surface area and 11 the average hernia defect width (24). At this point in time, the size of the hernia orifice derived from CT scans of the abdomen at rest and during Valsalva's maneuver can be used to analyze the critical resistance needed toward impacts related to pressure (CRIP). With increasing size, the CRIP of the reconstruction rises (2). In the future, the position of the hernia orifice should be added (3).

So far, computed tomography was mainly used to image complications of surgery or mesh repair (25). Here, we present evidence that the tissue elasticity, as a major influence on the durability of repair on the bench test, can be analyzed with the aid of an added Valsalva's maneuver in patients (4). In our opinion, the critical load is a worst-case scenario and should consider the area of instability of the abdominal wall rather than a single hernia orifice.

### Applying the GRIP Concept to the Individual Patient

Musculoskeletal dysfunction is a consequence of weaknesses of the abdominal wall and should be remedied by incisional hernia repair (26). A multitude of meshes are available, which can be placed in various abdominal planes using open, laparoscopic or robotic approaches (27). The recent years were characterized by the advent of new techniques in an effort to significantly reduce risk factors for recurrence (28–30). Many discussions imply biomechanical stresses at the mesh–tissue interface to be involved with seroma formation, wound healing impairment, bulging, or recurrence. The shakedown concept might provide a biomechanical theory to analyze these technical differences in more detail as has been shown in synthetic polymer compounds (31).

The surface interaction between mesh and tissues provides adhesion (32). The initial bonding process can be described as tackiness (33). The retention force at a given tackiness is increased by sutures, tacks, and glue. Information about the interaction between mesh surface, fixation elements, and tissue is important to the surgeon during the planning of the procedure. Our approach defines a load case for the planning of the repair. The load case was derived from previous work on static testing using round meshes with a diameter of 15 cm covering a round hernia orifice of 5 cm without fixation elements (34). With cyclic loading, a rapid dislocation of many meshes was observed under these conditions. With the need of additional fixation, we distinguished three different retention strength of the meshes called DIS classes A–C (8). The better the DIS class of a mesh, the less fixation is needed to take up and to reliably dissipate energy.

The retention strength of the fixation materials adds to the tackiness of the mesh. The increase is distinctly different between various materials (8). With information available from the DIS test, several surgical procedures have been successfully tested. So far, <200 out of about 4,500 surgical techniques have been analyzed. In the rapidly evolving market of hernia meshes with a significant potential for conflict, a premarket surveillance should include the mesh behavior as a compound (https://www.fda.gov/medical-devices/implants-and-prosthetics/hernia-surgical-mesh-implants&prev=search&pto=aue). This is important since more than 70 new hernia meshes were approved for clinical use by the FDA in recent years (35).

The change in the hernia size during pressure should be figured into the needed reconstruction strength in order to counteract the effects of tissue elasticity. In our group, a tissue factor as percent dilatation of the hernia size is multiplied with the hernia size to reach the required CRIP value preoperatively.

Intraoperatively, the regional distribution of tissue weaknesses or scar formations can be observed directly by the surgeon. As a consequence, the safety margin of the procedure might be elevated to reach higher GRIP values, or the distribution of fixation elements might be adjusted as desired. This was necessary in at least 12 reconstructions.

### Clinical Application of the Biomechanical Concept

Biomechanically stable repairs of ventral hernias result in low recurrence rates and low pain levels after 1 year (Figure 6). Biomechanically stable hernia meshes can be classified as DIS class A (8). If DIS class A meshes are augmented with sufficient fixation points and are implanted in sufficient size in the required layer of the abdominal wall, clinical results after 1 year are excellent (36).

For more than 80 years, biomechanically stable soft tissue repairs were modeled on the assumption that the strain of each constituent (cells, fibers, matrix, meshes, and fixation elements) equaled the global tissue strain resulting in an affine deformation (37). The results presented here prove that the models have to be extended to anisotropic and non-affine strain distributions. Recent work with finite element analysis provided important insights into the interaction of tissue and mesh (19). Even at low strain rates around 1 mm/min and during uniaxial stress, force accumulation at the suture fixation was observed. The methodology presented here provides a simple and clinically applicable way for the analysis of biomechanical stability during various conditions, such as multiaxial tension, anisotropic force distribution using dry or wet meshes with potentially wide variations of the experimental parameters, or standardized conditions as desired.

On the self-built bench test, about 85% of the combinations of meshes, defects, fixation materials, and closure defects tested failed before 425 DIS impacts. Since one third of our patients cough more often within the first 24 h after surgery, this might be one mechanism to explain failure rates and pain levels of the conventional approaches. The bench test results combined with CT scans of the abdominal wall of the individual patient enable the surgeon to enter a step-by-step assessment of the needs of the patient (Figures 3, 4). A durable repair has been observed after 1 year. The patients reported little or no pain. We plan to follow the patients for several years in the STRONGHOLD/Herniamed® registry.

A durable incisional hernia repair is sought after by many surgeons. One solution might be to use a high MDAR (38). A threshold of 16 was proposed by Hauters et al. (38) analyzing laparoscopic ventral hernia repair with DIS classes B and C meshes not taking into account fixation. Since the largest hernia meshes cover about 2,400 cm^2^, a ratio of 16 limits the hernia size to 150 cm^2^ to be durably repaired by this approach. For a round defect, a radius of 7 cm should not be surpassed according to these authors. A total of 21 patients reported here have hernia sizes above this limit. Our approach included the use of DIS class A mesh and fixation to increase the retention force.

Fixation can contribute to retention strength. An option may be to use an optimal ratio of fixation elements to the mesh area (39). Our approach uses a relative figure to tailor the reconstruction to the needs of the individual patient. As long as a threshold called CRIP is surpassed, there are many options to perform the repair. The study is limited by lacking information such as the minimal overlap related to the shape of the hernia orifice, by the low patient number, the limited observation period, and the lack of a control group.

Repair for giant, battlefield, recurrent, or complex incisional hernia can durably be performed according to the GRIP concept. With many new meshes and fixation devices in the regulatory process to gain market access, DIS testing can confirm a high DIS class of the material. The surgical algorithm provides a step-by-step approach. Stable repairs seem to heal without seroma formation or wound problems even in patients with many comorbidities (Tables 2, 3). The 1-year results of the first 96 patients operated on by 10 surgeons in four hospitals are encouraging for future developments.

## Clinical Implications and Imitations

The concept provides a biomechanical point of view to plan both individual incisional hernia repair and studies. At this point in time, only a small fraction of meshes, fixation devices, and glues have been tested. Many coefficients are still lacking as well as contributions from basic sciences. Is the overlap required for a durable repair a stable value, or does it vary with the type of mesh, the form of the hernia opening, and the instability of the abdominal wall? The work done so far will most likely look minute at the end, but it provides a starting point for the investigation of cyclic loading and critical load limits. Since this approach has been successful in compound constructions such as airplane wings, we confidently report our limited data and the first clinical application of a biomechanical-based incisional hernia repair.

## Conclusions

A bench test was designed delivering dynamic intermittent strain (DIS) similar to coughing impacts. A load limit was defined after counting coughs on patients postoperatively: one third of the patients coughed 425 times in 24 h or more often. Using the bench test with 425 DIS impacts as cyclic load, meshes, their position in the abdominal wall, fixation elements, repair techniques, and tissue elasticity were attributed to a relative figure called **g**ained **r**esistance to **i**mpacts related to **p**ressure (GRIP). With increasing defect size, GRIP is needed to be raised to reach durable repairs. The minimal GRIP required for a durable repair at a given size was defined as the **c**ritical **r**esistance to **i**mpacts related to **p**ressure (CRIP). We conclude that the GRIP needs to exceed the CRIP in the repair of large, recurrent, and complex incisional hernia.

Tissue elasticity in the individual patient was assessed with CT scans of the abdomen at rest and during Valsalva's maneuver. The hernia size was found to change upon pressure in about half of the patients up to 250 cm^2^. We conclude that the tissue elasticity and the hernia size under pressure should be assessed before the repair of large, recurrent, and complex incisional hernia.

Both the results from the bench test and from the CT scans gave the opportunity to develop a biomechanical basis for incisional hernia repair. The concept was applied by 10 surgeons in four hospitals. After 1 year, 96 consecutive patients were repaired using conventional techniques taking into account the biomechanical theory of the GRIP concept and the bench test and the CT results. No mortality, a complication rate of 5–7%, no recurrences, and low pain levels were observed. We conclude that the GRIP concept is a structured approach for the repair of large, recurrent, and complex incisional hernia.

## Data Availability Statement

The raw data supporting the conclusions of this article will be made available by the authors, without undue reservation.

## Ethics Statement

The studies involving human participants were reviewed and approved by Universität Heidelberg Ethikkommission der Med. Fakultät No. S-522/2020. Written informed consent for participation as required for this study was obtained in accordance with the national legislation and the institutional requirements.

## Author Contributions

FK and RN designed and directed all research, conducted some of the series, got the funding, received material support, were the key surgeons, drafted the report, and will do the revision. DG, FH, MS, YL, and RN investigated the tissues on the bench test. VL, TL, JR, and RN were key surgeons and applied the GRIP concept to patients. JG and AG did the CT scans and evaluated the results together with VL, RN, and FK. MV and FK designed the bench test. MV supervised the building process. All authors contributed to the article and approved the submitted version.

## Conflict of Interest

FK has received research grants from Baxter^®^, Dahlhausen^®^, Ethicon^®^, and Medtronic^®^ not related to the research perspective described in the manuscript. The remaining authors declare that the research was conducted in the absence of any commercial or financial relationships that could be construed as a potential conflict of interest.
